# Rapid Molecular Diagnosis of Sporotrichosis Directly from Biological Samples from a Reference Center in Brazil

**DOI:** 10.3390/jof10060432

**Published:** 2024-06-18

**Authors:** Amanda Gabriela da Silva, Arthur Felipe Cavalcanti de Matos, Bruna Rodrigues de Sousa, Claudia Elise Ferraz, Raul Leal Faria Luiz, Rejane Pereira Neves, Reginaldo Gonçalves de Lima-Neto, Manoel Marques Evangelista Oliveira

**Affiliations:** 1 Postgraduate Program in Tropical Medicine, Center for Medical Sciences, Federal University of Pernambuco (UFPE), Recife 50740-570, Pernambuco, Brazil; amanda.gabrielasilva@ufpe.br (A.G.d.S.); brunasousa14@hotmail.com (B.R.d.S.);; 2 Laboratory for Research and Diagnosis in Tropical Diseases, Federal University of Pernambuco (UFPE), Recife 50740-570, Pernambuco, Brazil; arthur.cavalcanti@ufpe.br; 3 Dermatology Reference Service, Hospital das Clínicas, Federal University of Pernambuco (UFPE), Recife 50670-901, Pernambuco, Brazil; claudiaelise@yahoo.com.br; 4 Oswaldo Cruz Institute, FIOCRUZ, Av Brasil, 4365, Manguinhos, Rio de Janeiro CEP 21045-360, Brazil; raulealuiz@gmail.com (R.L.F.L.); manoel.marques@ioc.fiocruz.br (M.M.E.O.); 5 Postgraduate Program in Fungal Biology, Biosciences Center, Federal University of Pernambuco (UFPE), Recife 50740-570, Pernambuco, Brazil

**Keywords:** *Sporothrix*, nested PCR, skin biopsy, skin exudate, ocular secretion

## Abstract

The gold standard diagnosis of sporotrichosis is the isolation of *Sporothrix* sp. in culture media, but this is a time-consuming test that is susceptible to contamination and can be affected by the fungal load. Molecular methods such as nested PCR are gaining more ground in the management of several infections as they are tools for the rapid and accurate identification of microorganisms from pure cultures or directly from biological samples. This study aimed to apply a nested PCR molecular protocol for the rapid detection of *Sporothrix* spp. directly from clinical samples. Thirteen samples—six from skin biopsies, five from skin exudates, and two from conjunctival secretions—were obtained from patients diagnosed with sporotrichosis due to *S. brasiliensis*. Calmodulin gene sequencing identified all the isolates as *S. brasiliensis*. Nested PCR was able to detect all the *Sporothrix* sensu lato directly from clinical samples as well as the CBS 120339 reference strain. The nested PCR protocol stands out as a diagnostic alternative, as it allows the identification of *Sporothrix* spp. directly from clinical samples without the need for fungal isolation.

## 1. Introduction

Sporotrichosis is a fungal disease with a global distribution that affects humans and animals. Most cases result from the traumatic inoculation of *Sporothrix* spp. propagules into the host’s skin tissue via animal or environmental transmission [1,2]. In Brazil, this infection has become a public health challenge due to the increase in its incidence in several states, especially in the northeastern region [1]. In the state of Pernambuco, Northeast Brazil, the fungus has been responsible for more than 900 cases in animals and 450 cases in humans over the last five years [3,4,5].

The current epidemic of sporotrichosis in Pernambuco is the result of the high stray cat population and poor sanitary conditions, which facilitate the spread of the fungus [4]. Up to now, the gold-standard diagnosis of sporotrichosis is the isolation of *Sporothrix* spp. from culture media. However, it is a time-consuming test that is susceptible to contamination, and the low fungal load in certain samples can hinder the growth of the fungus [6].

Delay in diagnosing this mycosis can lead to complications for the patient, such as longer antifungal therapy and a worsening of the disease. Thus, the use of molecular techniques has been gaining ground in the management of sporotrichosis, as they are tests based on the amplification of DNA fragments and are therefore able to quickly provide reliable results [7,8].

Among these methodologies, the use of nested PCR has emerged as a tool for the rapid and accurate identification of microorganisms from a few colony-forming units (CFU) of pure cultures and from samples of tissue fragments [8,9]. Nested PCR is a molecular method that generally involves two sequential amplification reactions and is used in situations where it is necessary to increase the sensitivity and/or specificity of the PCR [9]. There are no standardized nested PCR protocols for microbial identification directly from wound exudate and body secretions.

This study aimed to apply a molecular nested PCR protocol for the rapid detection and identification of *Sporothrix* spp. directly from clinical samples from patients followed at a sporotrichosis reference center in Northeast Brazil.

## 2. Methods

### 2.1. Sample Collection and Classical Mycological Diagnosis

Thirteen clinical samples from 12 patients were collected depending on the type of lesion, including six fragments of skin tissue, five exudates from cutaneous skin lesions, and two samples from conjunctival secretions. Tissue fragments were obtained with a dermatological punch (4 mm), and the exudate and conjunctival secretion samples were collected with sterile swabs pre-moistened with 0.9% sterile saline solution. All cases of sporotrichosis were followed and treated at the Dermatology Reference Service at the Hospital das Clínicas at the Federal University of Pernambuco between April and September 2023. Only patients with a diagnosis confirmed by the isolation of *Sporothrix* spp. by mycological culture were included. The fungus was isolated by sowing it onto Mycosel agar in Petri dishes incubated at 25–30 °C for up to 15 days.

A fragment of skin tissue from a patient positive for *Leishmania* spp. was used to evaluate specificity and the occurrence of cross-reactions. In addition, the DNA of a type strain of *S. brasiliensis* CBS 120339 (formerly IPEC 16490), was included as a positive control. To monitor possible contamination, reaction mixtures without DNA were performed in the nested PCR as a negative control.

### 2.2. DNA Extraction and Quantification from Clinical Samples and Strains

The DNA extraction for clinical samples was performed as described by Oliveira et al. [6], with modifications. Samples obtained through biopsy were macerated in a sterile saline solution. Then, 200 μL of each sample containing tissue fragment, lesion exudate, or conjunctival secretion was centrifuged for 5 min at 11,800× *g* to obtain pellets. DNA extraction was carried out using the QIAamp^®^ DNA mini kit (QIAGEN, Hilden, Germany) following the manufacturer’s instructions. After extraction, the extracted DNA was frozen at −20 °C. The DNA concentration was evaluated by spectrophotometric measurement of the absorbance at the 260 nm wavelength (NanoDrop™ 2000c, Thermo Fisher Scientific, Waltham, MA, USA).

DNA extraction from the strains was performed using the commercial Wizard^®^ Genomic DNA Purification kit. Briefly, fungal colonies were transferred to a DNA extraction tube containing glass beads, and then 300 μL of nuclear lysis solution was added. Afterward, the tubes were shaken at 5.5 m/s in a FastPrep instrument (BIO 101, Farmingdale, New York, NY, USA) and incubated at 65 °C for 15 min. Next, 100 μL of the protein precipitation solution was added, and then the mixture was shaken and centrifuged at 13,600× *g* for 5 min. The supernatant obtained was transferred to a new tube containing 400 μL of isopropanol. The new tube was homogenized by inversion and centrifuged at 13,600× *g* for 3 min. The supernatant was discarded, and 400 μL of 70% ethanol at room temperature was added and the sample centrifuged again. After centrifugation, the tube was inverted to completely remove the supernatant. The extracted DNA was resuspended in 50 μL of autoclaved Milli-Q water. DNA quantification was carried out on a NanoDrop™ 2000 (Thermo Fisher Scientific, Waltham, MA, USA).

### 2.3. Molecular Identification by Partial Sequencing of Calmodulin

Briefly, the amplification of the Calmodulin (CAL) genomic region was carried out as described by Lima-Neto et al. [10] using the CL1 sense (5′GA (GA) T (AT) CAA GGA GGC CTTCTC-3′) and CL2A antisense (5′-TT TTG CATCATGAGTTGGAC-3′) primers. The PCR was carried out in a Veriti™ 96-well thermal cycler (Thermo Fisher Scientific, Waltham, MA, USA) with annealing at 60 °C for 45 s. The amplicons were purified using the Wizard^®^ Genomic DNA Purification Kit (Promega Corporation, Madison, WI, USA).

The sequences obtained from the CAL gene sequencing were edited using the BioEdit™ software, version 7.2.6.1, and submitted to a search for similar sequences using the Basic Local Alignment Search Tool (BLAST). Phylogenetic analyses were carried out using our sequences and reference sequences. The sequences were aligned using the MAFFT v.7 online interface (available at https://mafft.cbrc.jp/alignment/server, and accessed on 28 February 2024) and edited with MEGA V.11 software (available at https://www.megasoftware.net/, accessed on 28 February 2024). *Grosmannia sersens* (CBS 141.36) was used as an outgroup.

### 2.4. Nested PCR for Clinical Samples

The assay was carried out according to the method based on the amplification of 18S ribosomal RNA previously described by Luiz et al. [8]. The reaction was carried out with a ready-to-use PCR master mix 2X solution (Promega Corporation, Madison, WI, USA—Lot: 22332002). A final concentration of 100 ng of DNA was used in a total reaction volume of 50 μL. In the first-round PCR, 10 μM concentrations of the outer primers SS1 (5’-CTCGTTCGGCACCTTACACG-3’) and SS2 (5’-CGCTGCCAAAGCAACGCGGG-3’) were used. The second-round PCR (nested PCR) was identical to the first-round PCR, except that 3 μL of the first reaction product and the internal primer pair SS3 (5’-ACTCACCAGGTCCAGACACGATG-3’) and SS4 (5’-CGCGGGCTATTTAGCAGGTTAAG-3’) were used. The PCR reaction was carried out according to the following conditions: 95 °C for 5 min, followed by 40 cycles at 95 °C for 1 min, 68 °C for 1 min, and 72 °C for 1 min, with a final extension at 72 °C for 10 min. PCR products were analyzed by electrophoresis on 2% *w*/*v* agarose gels, and DNA fragments were stained with Blue Green Loading Dye I (LGC Biotecnologia, São Paulo, Brazil—Lot: 160.919BT). The amplicons were visualized on a K33-333 LED transilluminator (KASVI, Paraná, Brazil). Furthermore, the time taken to perform this technique was calculated.

### 2.5. Ethical Aspects

The research was approved by the Human Research Ethics Committee (CEP) of the Hospital das Clínicas (HC/EBSERH/UFPE) under protocol CAAE: 71720323.8.0000.8807, as well as by the CEP of the Oswaldo Cruz Institute under protocol CAAE: 71720323.8.3003.5248.

## 3. Results

**Figure 1:**
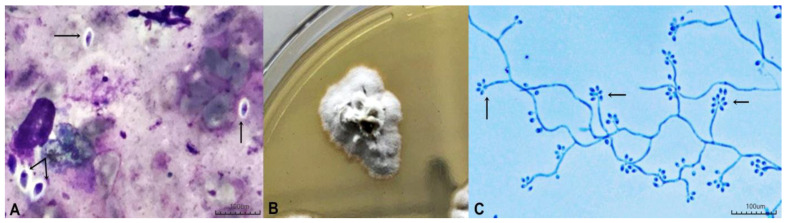
(**A**) Direct mycological examination showing yeast elliptical cells (arrows) with a panoptic halo; (**B**) *Sporothrix* spp. on Mycosel agar showing wrinkled, white colonies that gradually darkened to a blackish color; (**C**) Micromorphology, showing slender, septate mycelial filaments and conidiophores containing conidia with a “daisy-like” floral arrangement (arrow).

**Figure 2:**
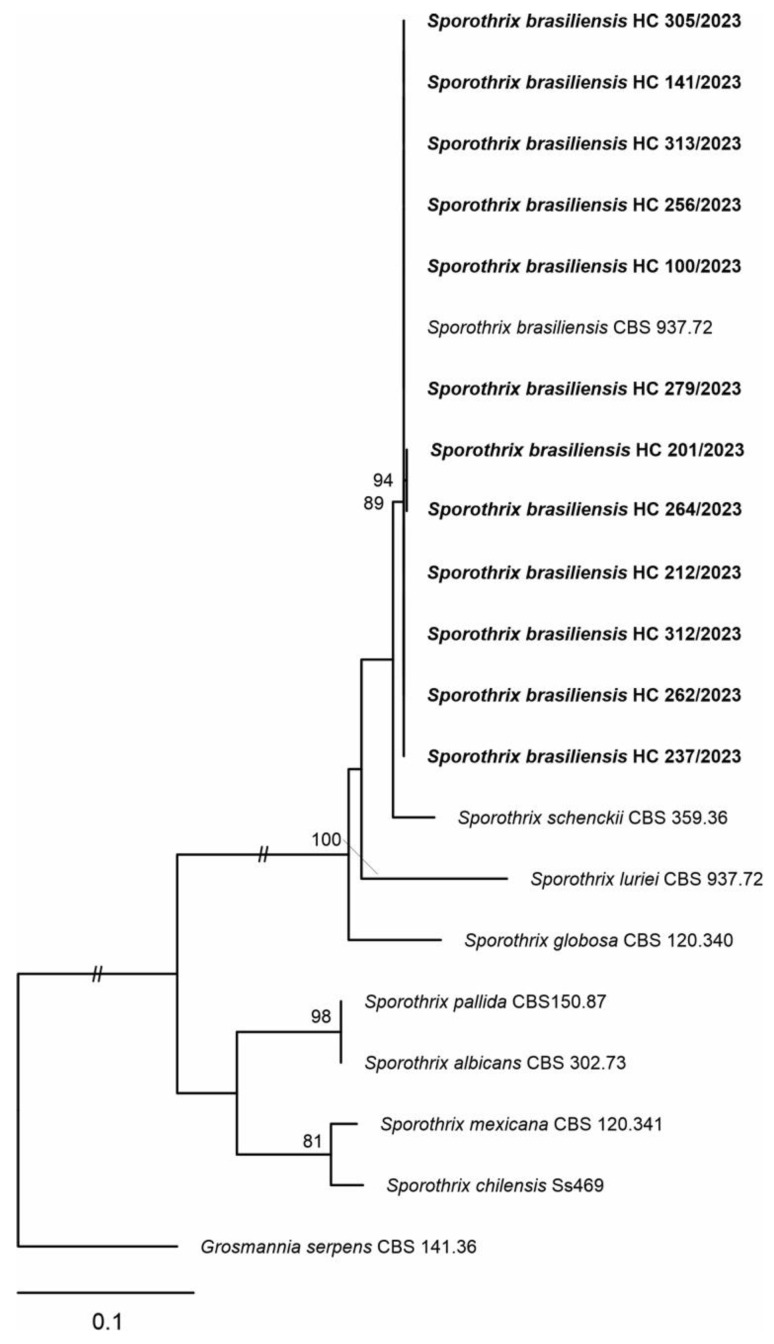
Phylogenetic tree of the genes encoding Calmodulin.

Direct examination was performed on seven samples (53.8%; n = 7/13). Among these, the presence of globular, oval, or elliptical yeast-like cells showing a halo following panoptic staining (Figure 1A) was observed in four samples. *Sporothrix* spp. cultures (Figure 1B) showed thin and hyaline septate mycelia with delicate conidiophores, finishing at an expanded denticulate vesicle at the apex and bearing the floral aspect (Figure 1C); these were recovered from Mycosel agar in 12 (92.3%) of the 13 samples included in the study. Only one sample of a skin fragment, from the patient who had two samples analyzed, was positive in mycological culture. Genotypic analysis by partial sequencing of the CAL gene was carried out on 12 isolated clinical strains of *Sporothrix*, identifying 100% of the isolates as *Sporothrix brasiliensis*, as shown in Figure 2 and summarized in Table 1.

**Figure 3:**
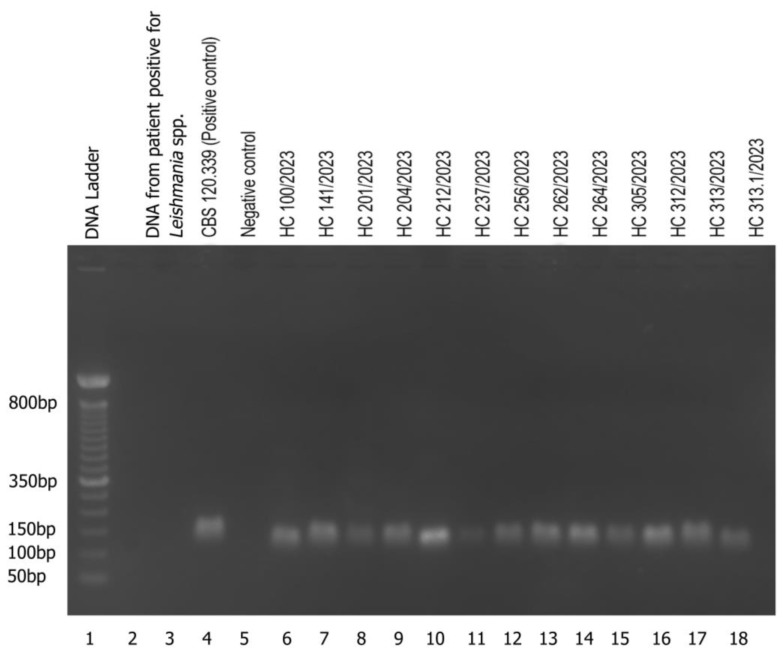
Nested PCR products on an agarose gel. From left to right: 1: Molecular marker DNA ladder, 50 bp (Invitrogen); 2: Empty well; 3: Cross-reaction evaluation with the *Leishmania* spp. sample; 4: Positive control (strain CBS 120339 (formerly IPEC 16490)); 5: PCR negative control (sterile water); 6: HC100/2023; 7: HC141/2023; 8: HC201/2023; 9: HC204/2023; 10: HC212/2023; 11: HC237/2023; 12: HC256/2023; 13: HC262/2023; 14: HC264/2023; 15: HC305/2023; 16: HC312/2023; 17: HC313/2023; 18: HC HC313. 1/2023. The nested PCR product is a 152 bp amplicon.

In this work, the molecular technique of nested PCR for the detection of *Sporothrix* spp. directly from clinical patient samples was evaluated for the first time in a dermatological reference center in the northeastern region of Brazil. The nested PCR methodology was able to detect the expected 152 bp fragment in all 13 clinical samples tested (Figure 3), suggesting the presence of *Sporothrix* sensu lato (Table 1). Given the absence of amplified product, no cross-reaction was observed in the nested PCR using the clinical sample of *Leishmania* spp. (Figure 3).

The time required to carry out the nested PCR protocol for each sample, from DNA extraction to the analysis of the PCR products, is described below: (1) DNA extraction = 20 min; (2) Quantification of DNA concentration and purity = 5 min; (3) DNA dilution = 5 min; (4) Preparation of the first PCR step = 10 min; (5) First PCR reaction = 135 min; (6) Preparation of the second PCR step = 10 min; (7) Second PCR reaction (Nested PCR) = 135 min; (8) Agarose gel electrophoresis = 120 min. The total procedure time was calculated to be 440 min (7.33 h).

The median age of the patients was 44.5 ± 16.2 years, with a predominance of males (58.3%; n = 7/12). Disseminated sporotrichosis was observed in 33.3% (n = 4/12) of the patients. Seven (58.3%; n = 7/12) patients reported having comorbidities, namely systemic arterial hypertension (SAH) in 57.1% (n = 4/7) and human immunodeficiency virus (HIV) in 42.9% (n = 3/7) of the patients. It is worth pointing out that all the patients with HIV developed the disseminated form of sporotrichosis. Contact with cats through scratching or biting was observed in nine (75.0%; n = 9/12) patients.

It is noteworthy that two clinical samples were obtained from one of the cases of disseminated sporotrichosis: one from a tissue fragment and the other from a skin exudate. *Sporothrix* spp. was isolated from the tissue fragment by mycological culture, whereas the fungus from the skin exudate sample did not grow in culture. Despite this, both samples were positive in the nested PCR protocol, as shown in Figure 3.

We assume that the nested PCR protocol applied presented 100% sensitivity due to the detection of *Sporothrix* DNA in all the samples, even in a culture-negative sample from a patient diagnosed with disseminated sporotrichosis. Considering that this protocol is capable of identifying the etiological agent as *Sporothrix* sensu lato, we also assume that it was 100% specific. However, the number of samples is small, and we cannot determine the accuracy.

Maximum likelihood (ML) analysis was carried out using RAxML-HPC BlackBox v. 8.2.12 on the CIPRES Science Portal (available at https://www.phylo.org/portal2/login, and accessed on 28 February 2024). The ML analysis was performed with 1000 bootstrap replicates, and values equal to or greater than 70% bootstrap ML support were shown next to the nodes. The resulting phylogenetic tree was visualized using the FigTree software version 1.4.4 (//tree.bio.ed.ac.uk/software/figtree/, accessed on 28 February 2024) and exported for editing.

## 4. Discussion

The standard reference method for diagnosing sporotrichosis is the isolation and identification of *Sporothrix* spp. in culture [11]. However, this is a time-consuming technique, requiring a few weeks for complete fungal identification [12]. Therefore, it is necessary to develop tests that are faster than mycological culture to identify the fungus. [13].

In the present study, our findings using the nested PCR technique proved to be a rapid diagnostic strategy, as it can be applied directly to clinical samples. While for mycological culture, a period of seven to thirty days is necessary for the growth and identification of the fungus, the nested PCR technique presented here made it possible to reach a diagnosis in around 7 h. Importantly, nested amplification of the 18S rRNA gene fragment may detect all *Sporothrix* species of the *Sporothrix* complex. Reference methods for species recognition are based on DNA sequences harbored in genomic loci that encode proteins such as Calmodulin [14,15,16]. In our set of clinical samples, it was possible to identify the presence of a species of *Sporothrix* sensu lato, i.e., *Sporothrix brasiliensis*, which is endemic in Brazil. However, we should point out that the target gene and the technique applied here may be widely used in areas where other species are more prevalent.

Orofino-Costa and collaborators (2022) reported in Brazilian guidelines dedicated to sporotrichosis that purulent samples and biopsies are the most suitable for diagnosing sporotrichosis [17]. Therefore, we included samples of tissue fragments, skin exudates, and conjunctival secretions in our experiments. We decided to include conjunctival secretions due to the emergence of cases of granulomatous conjunctivitis caused by *Sporothrix* spp. in northeastern Brazil [18]. Furthermore, to our knowledge, the nested PCR technique has never previously been used to detect *Sporothrix* DNA in an ocular clinical sample.

In our study, patients had a median age of 44 years old and were predominantly male. Sporotrichosis can affect people of any age, but adults are more affected due to their greater occupational and zoonotic exposure. In relation to gender, some studies have shown that women are more affected, as they are generally responsible for taking care of the cats at home and are more exposed to scratches and bites [4,11].

Clinically, sporotrichosis is a mycosis with manifestations ranging from skin lesions to disseminated forms. In general, the disseminated forms are related to some form of immunosuppression or secondary immunodeficiency, such as people living with HIV/AIDS. In this study, SAH was more common, but people living with HIV/AIDS were associated with three out of four cases of disseminated sporotrichosis, suggesting that one’s immunological condition influences the prognosis of the infection [17]. Co-infection between HIV and sporotrichosis has also been described in Rio de Janeiro. A retrospective analysis between 1999 and 2015 identified 75 hospital admissions for sporotrichosis, of which 38.7% (n = 29/75) involved people living with HIV/AIDS [19].

Despite the advantages of being able to do so, it is worth highlighting that few methods allow the detection of *Sporothrix* DNA directly from clinical samples [8,9,20]. Accordingly, nested PCR is a promising strategy because it can detect *Sporothrix* DNA not only in cultures but also in biological samples [9]. Studies have demonstrated that this molecular methodology has been used for genus-level diagnosis in formalin-fixed, paraffin-embedded (FFPE) tissue samples from patients with sporotrichosis and, more recently, in FFPE tissue samples from cats with sporotrichosis, showing a high level of sensitivity and specificity [8,20].

Our results suggest a high sensitivity of the method, since nested PCR was able to detect *Sporothrix* in a skin sample that did not show growth in culture. The absence of *Sporothrix* growth in culture can be explained by the low fungal load shown by DNA quantification. Similarly, other studies using nested PCR were able to detect *Sporothrix* in cerebrospinal fluid samples from patients even when it was not possible to recover the fungus from culture media. After identifying *Sporothrix* sensu lato in the tested samples, the authors stated that the method used is a new approach to diagnosing sporotrichosis and allows early treatment [6].

In hyperendemic areas such as Latin America, rapid and accurate diagnostic methods, such as the one presented in this study, are essentially necessary. This is because controlling sporotrichosis in these locations has become increasingly difficult. Thus, a fast and accurate diagnosis allows for a faster response from health agencies to control outbreaks [15]. However, to date, fast methodologies for the diagnosis of sporotrichosis, such as a point-of-care test, have not yet been developed.

## 5. Conclusions

In conclusion, our results suggest that the nested PCR protocol appears to be an alternative for the diagnosis of sporotrichosis that can be applied directly to clinical samples, eliminating the time-consuming isolation of the fungus by culturing. All positive clinical samples in the nested PCR showed the presence of *Sporothrix* species sensu lato. However, the identification of *Sporothrix* species directly from the DNA of the clinical sample depends on the development of new molecular strategies, especially in endemic areas with resource-limited settings, which can be the target of future studies. As a result, the early diagnosis and reliable treatment of patients are possible, especially in immunocompromised patients such as those in our sample.

## Figures and Tables

**Table 1:** Epidemiological and clinical laboratory findings of the patients diagnosed with sporotrichosis in a reference hospital in the state of Pernambuco, Brazil.

Sample ID.	AGE	SEX	Occupation	Clinical Form	Comorbidity	Sample Topography	Sample Type	Direct Exam *	Culture	Cal Gene	Nested PCR
HC100/2023	55	Fem.	Housekeeper	Parinaud oculoglandular syndrome	SAH	Left conjunctiva	Conjunctival secretion	Negative	*Sporothrix* spp.	*S. brasiliensis*	*Sporothrix* sensu lato
HC141/2023	64	Male	Freelancer salesman	Disseminated sporotrichosis	SAH	Left lower limb	Tissue fragment	Negative	*Sporothrix* spp.	*S. brasiliensis*	*Sporothrix* sensu lato
HC201/2023	44	Fem.	Housewife	Fixed sporotrichosis	SAH	Malar	Lesion exudate	Unrealized	*Sporothrix* spp.	*S. brasiliensis*	*Sporothrix* sensu lato
HC204/2023	37	Male	Programmer	Fixed sporotrichosis	Does not have	Right lower limb	Lesion exudate	Positive	*Sporothrix* spp.	*S. brasiliensis*	*Sporothrix* sensu lato
HC212/2023	48	Fem.	Housewife	Fixed sporotrichosis	Does not have	Right forearm	Tissue fragment	Unrealized	*Sporothrix* spp.	*S. brasiliensis*	*Sporothrix* sensu lato
HC237/2023	56	Male	Unemployed	Disseminated sporotrichosis	HIV	Conjunctiva	Conjunctival secretion	Unrealized	*Sporothrix* spp.	*S. brasiliensis*	*Sporothrix* sensu lato
HC256//2023	17	Male	Student	Not included	Does not have	Right hand	Tissue fragment	Unrealized	*Sporothrix* spp.	*S. brasiliensis*	*Sporothrix* sensu lato
HC262/2023	4	Fem.	Student	Parinaud oculoglandular syndrome	Does not have	Left conjunctiva	Lesion exudate	Unrealized	*Sporothrix* spp.	*S. brasiliensis*	*Sporothrix* sensu lato
HC264/2023	45	Male	Driver	Lymphocutaneous	SAH	Right forearm	Tissue fragment	Unrealized	*Sporothrix* spp.	*S. brasiliensis*	*Sporothrix* sensu lato
HC305/2023	47	Male	Unemployed	Disseminated sporotrichosis	HIV	Back	Tissue fragment	Positive	*Sporothrix* spp.	*S. brasiliensis*	*Sporothrix sensu lato*
HC312/2023	33	Male	Driver	Lymphocutaneous	Does not have	Right forearm	Tissue fragment	Negative	*Sporothrix* spp.	*S. brasiliensis*	*Sporothrix sensu lato*
HC313/2023	33	Fem.	Housewife	Disseminated sporotrichosis	HIV	Right forearm	Tissue fragment	Positive	*Sporothrix* spp.	*S. brasiliensis*	*Sporothrix sensu lato*
						Neck	Lesion exudate	Negative	Absence of fungal growth	Unrealized	*Sporothrix sensu lato*

Note: ID: Identifier; Fem.: Female; CAL: Calmodulin; SAH: Systemic arterial hypertension; HIV: Human Immunodeficiency Virus. * A positive direct mycological examination corresponds to the visualization of yeast cells presenting a halo upon panoptic staining.

## Data Availability

The original contributions presented in the study are included in the article, further inquiries can be directed to the corresponding author/s. The data about patients is unavailable due to ethical restrictions.
